# Differences in Factors Affecting Various Crash Types with High Numbers of Fatalities and Injuries in China

**DOI:** 10.1371/journal.pone.0158559

**Published:** 2016-07-20

**Authors:** Yikai Chen, Kai Wang, Mark King, Jie He, Jianxun Ding, Qin Shi, Changjun Wang, Pingfan Li

**Affiliations:** 1 School of Automotive and Transportation Engineering, Hefei University of Technology, Hefei, Anhui, China; 2 Traffic Management Research Institute of the Ministry of Public Security, Wuxi, Jiangsu, China; 3 Centre for Accident Research and Road Safety- Queensland, Queensland University of Technology (QUT), Brisbane, Queensland, Australia; 4 School of Transportation, Southeast University, Nanjing, Jiangsu, China; Chongqing University, CHINA

## Abstract

**Objectives:**

Road traffic crashes that involve very high numbers of fatalities and injuries arouse public concern wherever they occur. In China, there are two categories of such crashes: a crash that results in 10–30 fatalities, 50–100 serious injuries or a total cost of 50–100 million RMB ($US8-16m) is a “serious road traffic crash” (SRTC), while a crash that is even more severe or costly is a “particularly serious road traffic crash” (PSRTC). The aim of this study is to identify the main factors affecting different types of these crashes (single-vehicle, head-on, rear-end and side impact) with the ultimate goal of informing prevention activities and policies.

**Methods:**

Detailed descriptions of the SRTCs and PSRTCs that occurred from 2007 to 2014 were collected from the database “In-depth Investigation and Analysis System for Major Road Traffic Crashes” (IIASMRTC), which is maintained by the Traffic Management Research Institute of the Ministry of Public Security of China (TMRI). 18 main risk factors, which were categorized into four areas (participant, vehicle, road and environment-related) were chosen as potential independent variables for the multinomial logistic regression analysis. Comparisons were made among the single-vehicle, head-on, rear-end and side impact crashes in terms of factors affecting crash occurrence.

**Findings:**

Five risk factors were significant for the six multinomial logistic regression models, which were location, vertical alignment, roadside safety rating, driver distraction and overloading of cargo. It was indicated that intersections were more likely to have side impact SRTCs and PSRTCs, especially with poor visibility at night. Overloaded freight vehicles were more likely to be involved in a rear-end crash than other freight vehicles. Driver distraction is an important risk factor for head-on crashes, while vertical alignment and roadside safety rating are positively associated with single-vehicle crashes.

**Conclusion:**

Based on the findings, promising measures were proposed to prevent each type of SRTC and PSRTC, which governmental or regulatory agencies could employ to plan strategies to reduce SRTCs and PSRTCs and support lifesaving policies.

## Introduction

The global burden of road crashes has been rising with increasing motorization, and it is estimated that there are over 1.2 million people killed and up to 50 million people injured in road traffic crashes each year [1]. While the cost of these crashes is hard to calculate with certainty, it is considered to be between 1% and 3% of a country’s Gross Domestic Product. Across the world, this amounts roughly to 500 billion US dollars, a substantial figure. Although non-fatal collisions are more numerous, fatal crashes—and especially crashes with high numbers of fatalities and injuries—have much more significant impacts on those involved and their families. The costs of medical care, loss of income (often due to death or injury of the main breadwinner) and demands of care for long term impairment can be impoverish families.

The “493 Decree” published by the China State Council contains definitions for road traffic crashes with very high numbers of fatalities and injuries or a high cost. A “serious road traffic crash” (SRTC) is defined as one where 10 to 30 people die in the crash, or 50 to 100 people are seriously injured, or the crash leads to direct economic loss of 50 to 100 million RMB (about 8 to 16 million US dollars). A “particularly serious road traffic crash” (PSRTC) is more costly, involving more than 30 deaths, or more than 100 seriously injured people, or more than 100 million RMB direct economic loss. Official statistics for the period 2007 to 2014 show that 2908 deaths and 2897 injuries occurred in 194 SRTCs and PSRTCs [2]. Most of these crashes (187) are SRTCs; the 7 PSRTCs accounted for 294 deaths and 69 injuries.

Prediction models are a well-recognized means of analyzing the factors that contribute to an increased road crash risk, and therefore could be addressed through preventive measures. However, what constitutes an efficient and effective prediction model can vary depending on specific objectives such as what is being predicted and at which level [3]. The majority of prediction models attempt to predict crash severity. These models are divided into 3 major classifications: (1) binary outcome models, which are typically applicable to situations with dichotomous injury severity outcomes, such as fatal and nonfatal crashes; (2) ordered discrete outcome models, which account for the ordinal nature of injury data (e.g., ranging from property damage only crashes to injury crashes and fatal crashes); and (3) unordered multinomial discrete outcome models, which take into account three or more outcomes and do not specifically consider the ordering of injury severity data [4]. In addition to crash severity, a considerable number of researchers have aimed at predicting frequency (number of crashes that occurred during a period) or crash rate (crash frequency relative to traffic exposure). Common methods include Poisson regression models, negative binomial regression models, and variations of these models [5].

Despite the contribution of previous modeling exercises, it has been argued in recent studies that the nature and hazardous factors influencing crash occurrence vary significantly by crash type, e.g. single-vehicle, head-on, etc. [6, 7]. Thus, identifying risk factors for different crash types is of great importance in promoting an understanding of various crash occurrence mechanisms and informing consequent prevention activities and policies. Golob and Recker applied nonlinear canonical correlation together with cluster analyses to more than 1000 crashes in Southern California to determine how crash type (e.g., rear-end, sideswipe, hit object) was related to traffic flow conditions [8]. Sullivan and Daly employed descriptive statistics to compare crashes with and without median trees with regard to crash types based on crash data on urban and suburban conventional highways in California [9]. It was found that median trees were associated with lower proportions of head-on and broadside collisions and with a higher proportion of hit-pedestrian collisions. In addition, more single vehicle collisions and fewer collisions involving three or more parties occurred in the presence of median trees. In Basyouny’s study [10], the relationship between the combination of different weather elements and seven crash types was explored using multivariate Poisson lognormal (MVPLN) models based on 11 years of daily weather and crash data for Edmonton, Canada. Adverse weather states were found associated with an increase of 9% to 73.7% for all crash types, with the highest increase for run-off-the-road crashes. The effects of more comprehensive factors (human-related, road-related, vehicle-related, traffic related factors, etc.) on the occurrence of specific crash types were considered in Yu and Larsen’s research [11, 12]. Active traffic management strategies designed for traffic safety improvement were proposed based on the identification of the real-time crash patterns.

The literature mentioned above demonstrates that most crash-type analyses have been undertaken in developed countries and regions. However, the characteristics and mechanisms of crashes in developing countries are different from those in developed countries [13–15]. In addition, road traffic crashes with high numbers of fatalities and injuries are much more frequent in developing countries, e.g. in China. Investigations into such crashes are of great importance in saving lives and meeting public expectations of safety. Therefore, the purpose of this study is to bridge the research gap on differences among SRTCs and PSRTCs in China by studying the factors affecting the occurrence of crash types (single-vehicle, head-on, rear-end and side impact), based on data on SRTCs and PSRTCs that occurred from 2007 to 2014. Moreover, an effort is made to highlight the preventive measures and policies for each crash type.

## Materials and Methods

### Data Preparation

In China, every SRTC and PSRTC is investigated thoroughly by the Ministry of Public Security (MPS)’s expert team with all the data and files recorded in a comprehensive database—In-depth Investigation and Analysis System for Major Road Traffic Accidents (IIASMRTA). The expert team is composed of local traffic police authorities, road traffic crash inspectors of the MPS and university professors with relevant expertise, and risk factors are identified by them for each SRTC and PSRTC based on the combination of witness interviews, hospital records, coroners’ reports, physical evidence and collision reconstruction. The risk factors in this study were selected from two sources: the “Traffic Violation Code Table” published by the MPS and the “Relevant Factors” listed in the IIASMRTA database. The former source is related to traffic violation behaviors, and the latter source classifies factors into three categories: participant-related factors (contains four subcategories: distraction, health problem, misjudgment, operation error); vehicle-related factors; and road and infrastructure-related factors.

In this study, data for all 194 SRTCs and PSRTCs that occurred in China from 2007 to 2014 were collected from the IIASMRTA database with the assistance of the Traffic Management Research Institute of the Ministry of Public Security of China (TMRI). A preliminary data screening procedure developed by Jiang and Lyles was followed to make the crash data relatively error free [16]. Five crashes were filtered out as a result of missing information and the final data contained 189 crashes, which comprised 100 single-vehicle crashes, 39 head-on crashes, 26 rear-end crashes and 24 side impact crashes (side crashes and sideswipe crashes). The percentage of missing records (2.6%) was much lower than those in several similar studies [4, 17, 18], which implies relatively good data quality.

The validity and reliability of the assigned risk factors for the crashes were cross-checked by the authors, through an examination of witness interviews, hospital records, and coroners reports and local police reports. This was undertaken for only part of the period (2007 to 2011) with the assistance of TMRI. A second cross-checking approach was undertaken by the first author through on-the-spot investigations of three SRTCs in 2012/13. The first author accompanied the expert team when they attended the crashes immediately after their occurrence, analyzed the same sources of evidence and reconstruction used by the expert team, and as a result was able to validate the risk factors assigned by the team.

The risk factors were ranked in terms of overall frequency of involvement in crashes, and the top 18 factors were chosen as potential independent variables for multinomial logistic regression. These factors were chosen because they met the requirement of data size for multinomial logistic regression analysis, which is a minimum of 10 cases per independent variable [19]. Other factors with lower ranks did not meet the requirement. The 18 factors can be divided into four categories (participant-related, vehicle-related, road-related, environment-related) [20, 21]. Table 1 provides the summary descriptive statistics for the dependent and potential independent variables used in this paper.

**Table 1:** Summary of variables and descriptive statistics.

Number	Variables	Types	Coding	Descriptive Statistics
	**Dependent variable**			
1	Crash Type	Nominal	1 = Single-vehicle; 2 = Head-on; 3 = Rear-end; 4 = Side impact	52.9% (n = 100); 20.6% (n = 39); 13.8% (n = 26); 12.7% (n = 24)
	**Potential independent variables**			
2	Location	Binary	0 = Intersection; 1 = Non-intersection	5.8% (n = 11); 94.2% (n = 178)
3	Horizontal alignment	Binary	0 = Curved; 1 = Straight	52.4% (n = 99); 47.6% (n = 90)
4	Vertical alignment	Binary	0 = Sloping; 1 = Flat	64.0% (n = 121); 36.0% (n = 68)
5	Lane width	Continuous	Lane width (m)	Mean = 3.54; Std Dev. = 1.01
6	Median strip	Binary	0 = Missing or nonstandard1 = Otherwise	7.4% (n = 14) 92.6% (n = 175)
7	Roadside safety infrastructure	Binary	0 = Missing or nonstandard; 1 = Otherwise	60.8% (n = 115); 39.2% (n = 74)
8	Signs	Binary	0 = Missing or nonstandard; 1 = Otherwise	56.1% (n = 106); 43.9% (n = 83)
9	Markings	Binary	0 = Missing or nonstandard; 1 = Otherwise	47.6% (n = 90); 52.4% (n = 99)
10	Roadside safety rating	Binary	0 = Hazardous and not traversable; 1 = Otherwise	57.7% (n = 109); 42.3% (n = 80)
11	Road adhesion condition	Binary	0 = Slippery; 1 = Dry	29.6% (n = 56); 70.4% (n = 133)
12	Lighting condition	Binary	0 = Dark with no supplemental street lights; 1 = Otherwise	30.2% (n = 57); 69.8% (n = 132)
13	Speeding	Binary	0 = Yes; 1 = No	67.2% (n = 127); 32.8% (n = 62)
14	Illegal overtaking	Binary	0 = Yes; 1 = No	10.1% (n = 19); 89.9% (n = 170)
15	Fatigued driving	Binary	0 = Yes; 1 = No	11.6% (n = 22); 88.4% (n = 167)
16	Driver distraction	Binary	0 = Yes; 1 = No	38.6% (n = 73); 61.4% (n = 116)
17	Overloading of people	Binary	0 = Yes; 1 = No	53.4% (n = 101); 46.6% (n = 81)
18	Overloading of cargo	Binary	0 = Yes; 1 = No	16.4% (n = 31); 83.6% (n = 158)
19	Vehicle malfunction	Binary	0 = Yes; 1 = No	41.8% (n = 79); 58.2% (n = 110)

Note: Percentages are provided for the nominal/ binary variables; mean and standard deviation values are provided for the continuous variables.

The regulations that apply to some of the road-related and participant-related risk factors listed in Table 1 are different from those in other countries, thus, some explanation of these factors is needed. Regarding “Missing or nonstandard median strip”, the type, width, and barrier specification of median strips on highways and urban roads with various grades are regulated by highway and road design standards, nevertheless, substandard median barriers or missing median strips are found in some SRTCs and PSRTCs. Such road deficiencies tend to cause crashes in which vehicle(s) deviate into the opposite lane and encounter a head-on collision with other vehicle(s). With respect to the factors “Missing or nonstandard signs” and “Missing or nonstandard markings”, although the Ministry of Transport has issued a series of standards regarding the design and use of signs or markings, missing warning signs before hazardous locations (e. g. road sections next to slopes/embankments and bodies of water, or with a high roadbed, or situated at a combination of horizontal curve and steep slope) are very common. Missing or vague road markings (e. g. lane demarcation lines, lane border lines, delineators, shoulder rumble strips) are also frequently observed near or at such locations.

In participant-related factors, “Illegal overtaking” is defined as “overtaking that occurs in overtaking-prohibited road sections or without safe overtaking conditions”. “Fatigued driving” is referred as “continuous driving over 4 hours without a break or a break time less than 20 minutes”. “Driver distraction” is explained as “the diversion of driver attention away from the driving task, which includes passenger-related distraction, cell phone-related distraction, cognitive distraction, and in-vehicle distraction”.

### Methods

The main objective of this research is to identify factors that have a significant influence on the occurrence of each crash type. Multinomial logistic regression is employed for two reasons: firstly, the impact of factors can be analyzed quantitatively since this method is capable of predicting the probability of each category of the dependent variable based on multiple independent variables. The results thus become more explicable than qualitative methods, e. g. decision tree. Secondly, multinomial logistic regression does not require the normality, linearity, or homoscedasticity of the data, so it is more attractive than methods which necessitate more assumptions about the data, e.g. discriminant function analysis [22].

However, multinomial logistic regression does need careful consideration of the correlation between each independent variable and the dependent variable (crash type), as well as the possible multicollinearity among the independent variables.

Since Pearson’ chi-square test is suitable for correlation analysis between two categorical variables, it was used to evaluate the relationship between each potential independent variable and crash type [23]. Independent variables with *P* values greater than 0.05 were eliminated from the subsequent analyses. The formula of the Pearson’ chi-square test statistic is [24]

$$\chi^{2}=\sum_{i=1}^{n}\frac{{\left(O_{i}-E_{i}\right)}^{2}}{E_{i}}=N\sum_{i=1}^{n}\frac{{\left(O_{i}/N-p_{i}\right)}^{2}}{p_{i}}$$ (1)

Where *χ*^2^ is the Pearson cumulative test statistic; *O*_*i*_ is the number of observations of type *i*; *N* is the total number of observations; *n* is the number of cells for *N* observations; *E*_*i*_ is the expected (theoretical) frequency of type *i*, which is expressed as

$$E_{i}=np_{i}$$ (2)

Where *p*_*i*_ is the fraction of type *i* in the population. *χ*^2^ is used to calculate a *P-value* by comparing the value of the statistic to a chi-squared distribution. The number of degrees of freedom) is equal to *n* minus the reduction in degrees of freedom.

After conducting Pearson’ chi-square test, bivariate correlation analysis was undertaken to identify the potential correlations among the remaining variables. Since most variables are discrete, Spearman’s rank correlation coefficients rather than Pearson’s correlation coefficients was used [25]. The Spearman’s rank correlation coefficient is given by [26]

$$r_{s}=1-\frac{6\sum_{i=1}^{\text{m}}d_{i}^{2}}{m^{3}-m}$$ (3)

Where *d*_*i*_ is the difference between ranks for each *x*_*i*_, *y*_*i*_ data pair and *m* is the number of data pairs.

Subsequently, a multinomial logistic regression model was employed to reveal the relationships among single-vehicle, head-on, rear-end and side impact crashes. Multinomial logistic regression is used to predict the probability of category membership of a dependent variable based on multiple independent variables. In this paper, the model is expressed as [27]

$$\text{log}\left(\frac{\pi_{i}}{\pi_{j}}\right)=\alpha_{i}+x^{T}\beta_{i}$$ (4)

Where *π*_*i*_ is the probability of non-baseline category *i* of the response variable (crash type), *i* = 1,*…p* (*i*≠*j*); *p* is the number of categories of the response variable; *π*_*j*_ is the probability of baseline category *j* of the response variable; *α*_*i*_ is the intercept of the *i*-th equation; **X**^**T**^ is the transpose of the independent variable vector **x**; **β**_**i**_ is the coefficient vector for *i*-th equation.

Finally, the statistical significance of the variables and the fitness of the model were assessed [28]. All the statistical analysis was performed in SPSS version 22 statistical package for Windows.

## Results

Table 2 shows the results of Pearson’s chi-square tests. The results indicate that several independent variables are not statistically significant at the 0.05 level, including “Speeding”, “Fatigued driving”, “Overloading of people” and “Vehicle malfunction”. Thus these variables were discarded.

**Table 2:** Pearson’s chi-square test results.

Number	Potential independent variables	Chi-square Statistic	*P*-value
2	Location	24.450	0.000
3	Horizontal alignment	38.010	0.000
4	Vertical alignment	45.364	0.000
5	Lane width	23.509	0.000
6	Median strip	12.754	0.005
7	Roadside safety infrastructure	51.765	0.000
8	Signs	12.454	0.006
9	Markings	27.911	0.000
10	Roadside safety rating	83.711	0.000
11	Road adhesion condition	7.871	0.049
12	Lighting condition	11.767	0.008
13	Speeding	0.955	**0.812**
14	Illegal overtaking	9.294	0.026
15	Fatigued driving	7.688	**0.053**
16	Driver distraction	25.338	0.000
17	Overloading of people	6.769	**0.080**
18	Overloading of cargo	48.072	0.000
19	Vehicle malfunction	3.179	**0.365**

Table 3 gives the Spearman’s rank correlation coefficients among the remaining factors; many strong correlations are observed at a 0.01 significance level.

**Table 3:** Spearman’s correlation coefficient matrix.

**Variable number**	**2**	**3**	**4**	**5**	**6**	**7**	**8**	**9**	**10**	**11**	**12**	**14**	**16**	**18**
2	—	-0.261**	-0.237**	0.014	0.016	-0.078	-0.008	0.034	-0.199**	-0.112	0.132	-0.083	0.174*	0.195
3		—	0.610**	0.361**	-0.297**	0.429**	0.288**	0.400**	0.448**	0.201**	-0.158*	-0.139	-0.070	-0.236**
4			—	0.267**	-0.251**	0.325**	0.203**	0.295**	0.473**	0.173*	-0.108	-0.189**	-0.107	-0.085
5				—	-0.151*	0.341**	0.315**	0.343**	0.305**	-0.034	-0.280**	-0.155*	0.019	-0.166**
6					—	-0.187**	-0.157*	-0.189**	-0.167*	-0.095	0.078	0.174*	-0.058	0.093
7						—	0.426**	0.526**	0.717**	0.093	-0.300**	-0.092	-0.121	-0.347**
8							—	0.566**	0.342**	0.014	-0.208**	-0.059	-0.086	-0.213**
9								—	0.367**	0.031	-0.234**	-0.072	-0.038	-0.165**
10									—	0.181*	-0.254**	-0.141	-0.200**	-0.315**
11										—	-0.073	0.091	-0.182*	-0.131
12											—	-0.105	0.071	0.114
14												—	-0.012	-0.006
16													—	0.177*
18														—

*P < 0.05.

**P < 0.01.

In order to eliminate multicollinearity among the factors, the “Forward Selection (Likelihood Ratio)” stepwise selection method was employed in the multinomial logistic regression analysis, with variable entry testing based on the significance of the score statistic (the significance level was set at *P*≤0.05), and removal testing based on the probability of a likelihood-ratio statistic based on the maximum partial likelihood estimates (the significance level was set at *P*>0.10) [29]. Finally, only five factors remained (shown in Table 4). The likelihood ratio test shows that all of them are statistically significant at a 0.05 level of significance.

**Table 4:** Likelihood ratio test.

	-2 Log likelihood of reduced model	Chi-square	Degrees of freedom	Significance
Intercept	275.715	.000	0	.
Location	298.784	23.069	3	0.000
Vertical alignment	293.332	17.617	3	0.001
Road side safety rating	311.542	35.827	3	0.000
Driver distraction	299.084	23.369	3	0.000
Overloading of cargo	310.438	34.723	3	0.000

Outcomes of the multinomial logistic regression model are used to predict the odds that the dependent variable (crash type) will be in one category as compared to another category. Thus, for this analysis, there are six category contrasts for the crash types: (1) single-vehicle crashes compared to side impact crashes, (2) head-on crashes compared to side impact crashes, (3) rear-end crashes compared to side impact crashes, (4) single-vehicle crashes compared to head-on crashes, (5) single-vehicle crashes compared to rear-end crashes, (6) head-on crashes compared to rear-end crashes. The output of a multinomial logistic regression model with four categories of dependent variable usually reveals three relationship contrasts, and this is typically how the results of such analyses are reported. However, all six of the relationship contrasts are provided to offer the reader additional insights that may not have been easy to determine otherwise [30]. Table 5 summarizes the parameter estimates of the final models, it should be noted that the reference level for each binary variable in SPSS is 1 rather than 0.

**Table 5:** Estimation results of multinomial logistic regression model.

**Variable**	**Single-vehicle crashes vs side impact crashes**			**Head-on crashes vs side impact crashes**			**Rear-end crashes vs side impact crashes**		
	Estimate	S. E	Odds ratio (95% CI)	Estimate	S. E	Odds ratio (95% CI)	Estimate	S. E	Odds ratio (95% CI)
Intercept	.291	.590		1.114	0.541		0.376	0.605	
Location	-.547	1.104	0.579 (0.066, 5.036)	-3.922	1.240	0.020 (0.002, 0.225)	-22.355	0.000	1.957E-10 (1.957E-10, 1.957E-10)
Vertical alignment	1.536	.671	4.647 (1.248, 17.312)	-0.922	0.671	0.398 (0.107, 1.481)	0.321	0.718	1.379 (0.338, 5.628)
Roadside safety rating	1.872	.662	6.504 (1.778, 23.792)	-1.188	0.689	0.305 (0.079, 1.177)	-1.218	0.740	0.296 (0.069, 1.262)
Driver distraction	-1.934	.635	0.145 (0.042, 0.502)	0.759	0.625	2.137 (0.627, 7.275)	-0.244	0.671	0.783 (0.210, 2.921)
Overloading of cargo	-3.786	1.248	0.023 (0.002, 0.262)	0.482	0.767	1.619 (0.360, 7.283)	1.456	0.768	4.289 (0.951, 19.335)
**Variable**	**single-vehicle crashes compared to head-on crashes**			**single-vehicle crashes vs rear-end crashes**			**head-on crashes vs rear-end crashes**		
	Estimate	S. E	Odds ratio (95% CI)	Estimate	S. E	Odds ratio (95% CI)	Estimate	S. E	Odds ratio (95% CI)
Intercept	-0.823	0.501		-0.086	0.570		0.738	0.494	
Location	3.374	1.559	29.205 (1.374, 620.573)	20.807	1.104	1087698862 (124953839.6, 9468206977)	17.433	1.240	37243762.62 (3277046.466, 423276834.4)
Vertical alignment	2.459	0.626	11.688 (3.427, 39.862)	1.215	0.667	3.370 (0.911, 12.462)	-1.244	0.589	0.288 (0.091, 0.915)
Roadside safety rating	3.060	0.647	21.333 (6.006, 75.770)	3.091	0.698	21.990 (5.603, 86.308)	0.030	0.699	1.031 (0.262, 4.059)
Driver distraction	-2.693	0.636	0.068 (0.019, 0.235)	-1.690	0.675	0.185 (0.049, 0.692)	1.003	0.563	2.727 (0.905, 8.215)
Overloading of cargo	-4.268	1.257	0.014 (0.001, 0.165)	-5.242	1.217	0.005(0.000, 0.057)	-0.974	0.593	0.377 (0.118, 1.207)

Note: Goodness-of-fit-statistics: Deviance = 272.52; Degrees of freedom = 513; *P* = 1.000.

−2Loglikelihood: The initial model only with the constant: 449.848; The final model: 275.715.

Pseudo-R^2^: Cox & Snell = 0.602; Nagelkerke = 0.662; McFadden = 0.385.

Overall prediction accuracy: 75.1%.

The quality of the model was examined. Firstly, the result of the goodness-of-fit test indicates that deviance P-values are greater than 0.05, which means that the model has acceptable fitness [5]. Compared with the model with only the intercept, the change in −2Loglikelihood in the final model is 174.133, which is well above the critical value at a 0.05 significance level for 15 degrees of freedom and implies that the model has a sufficient explanatory power [31]. In addition, the values of pseudo-R^2^ indicate a reasonable level of fit, and the overall prediction accuracy is desirable (75.1%) compared with previous literature [32–34].

## Discussion

Taking side impact crash as the reference type, it is shown in Table 3 that the signs of the “Location” variable are consistently negative for all three models (single-vehicle vs side impact, head-on vs side impact, rear-end vs side impact), which indicates that intersections are more likely to have side impact SRTCs and PSRTCs. This phenomenon can be understood as traffic lights are sometimes missing or malfunctioning at intersections in China. Therefore, drivers may fail to notice a vehicle coming from their side, especially at night with poor visibility. In the data set, eight side impact SRTCs and PSRTCs occurred at intersections, all traffic lights were missing or malfunctioning, and six of the crashes occurred at night without street lights. This finding is also generally in line with Abdel-Aty et al’s suggestion that side impact crashes have increased injury severity compared with other crash types at intersections [35].

When rear-end crashes are considered as the reference type, it is found that “Location” has positive signs for all the models, which means rear-end crashes are less likely to occur at intersections. Similarly, the positive coefficients of “Roadside safety rating” means that rear-end crashes are less likely than other crashes to happen at locations with hazardous and non-traversable roadsides (e.g. locations next to slopes/embankments and bodies of water or with a high roadbed). The coefficients of “Overloading of cargo” are all negative, thus, freight vehicles are more likely to be involved in a rear-end crash when overloaded. A reasonable explanation of this phenomenon is that overloading of freight vehicles tends to cause brake failure and increases braking distance [36], which in turn leads to rear-end collision with vehicles in front. Support for this interpretation can be found in crash information that shows that brake failure occurred in 11 of 13 “Overloading of cargo” rear-end crashes.

Roads with vertical grades are more likely to have single-vehicle crashes (OR = 11.688), rear-end crashes (OR = 3.472) and side impact crashes (OR = 2.513) when compared to head-on crashes. “Driver distraction” has negative signs for the three models, which demonstrates that distracted drivers are more likely to be involved in a head-on crash. The main reason for this kind of crash is inattentive driving or overtaking other vehicle(s) dangerously, and crossing the centerline without noticing the on-coming vehicle [23]. Suitable preventive measures include appropriate road markings and the posting of overtaking-forbidden signs, and the installation of caution signs/devices such as centerline rumble strips to raise drivers’ awareness at road locations prone to head-on-crashes.

For single-vehicle SRTCs and PSRTCs, the coefficients of “Driver distraction” and “Overloading of cargo” are positive, demonstrating that they are not the main risk factors for such crashes. However the negative signs of “Vertical alignment” and “Roadside safety rating” mean they are highly associated with single-vehicle crashes. There are in total 100 single-vehicle crashes, 76 of which occurred at locations with vertical grades and hazardous and non-traversable roadsides. It is worth noting that 73 are run-off-road crashes with lack of, or nonstandard, roadside safety infrastructure. As provision of clear zones is usually not an economical option for these road sections, roadside barriers of the proper type and sufficient strength are strongly suggested. Delineators, shoulder rumble strips, and warning signs that remind drivers of danger should also be implemented [37].

## Conclusions

Eighteen main risk factors of the SRTCs and PSRTCs that occurred from 2007 to 2014 were derived from the IIASMRTC database, which is maintained by the TMRI. Multinomial logistic regression analysis was performed to compare single-vehicle, head-on, rear-end and side impact crashes in terms of factors affecting crash occurrence. Based on the analysis results, several important findings and recommendations can be made:

1. Intersections are more likely to have side impact SRTCs and PSRTCs, especially those with poor visibility at night. Maintenance of traffic lights and street lights would be feasible measures to decrease such crashes.
2. Freight vehicles are more likely to be involved in a rear-end crash when overloaded. Since the major motivation for overloading of freight vehicles is the excessive pursuit of financial benefit, increasing regular checks by traffic police and improving transportation management to increase the income of contractors and drivers are practical ways to prevent overloading of cargo.
3. Distracted drivers are more likely to be involved in a head-on crash. The main reason for this kind of crashes is inattentive driving or overtaking other vehicle(s) dangerously, and crossing the centerline without noticing the on-coming vehicle. Promising preventive measures include appropriate road markings and the posting of overtaking-forbidden signs, and the installation of caution signs/devices such as centerline rumble strips to raise drivers’ awareness at head-on-crashes-prone road locations.
4. Vertical alignment and roadside safety rating are highly associated with single-vehicle crashes in a positive way, and most are run-off-road crashes. As provision of clear zones is usually not an economical option for these road sections, roadside barriers of the proper type and sufficient strength are strongly suggested. Delineators, shoulder rumble strips and warning signs that remind drivers of danger should also be implemented.

Despite the contributions of this work, a limitation lies in the validity and reliability of the risk factors. The risk factors collected from the IIASMRTA database were determined through in-depth investigation and analysis by the expert team. Although the authors reviewed the same evidence for most of the period under consideration, and validated the expert team assignment factors in three incidents through on-the-spot investigation, the reliability of the factors assigned across all the cases cannot be completely guaranteed.

Another limitation is the size of the data set. Information about SRTCs and PSRTCs that occurred before 2007 was unavailable in the IIASMRTA database and thereby not analyzed in this study. Although the data set used in this study met the desired sample size, a larger sample size would increase the prediction accuracy of the model and enable the authors to investigate more potential contributing factors at various significance levels [38], which should be focused on in the future.
